# Mechanisms of Antisense Transcription Initiation with Implications in Gene Expression, Genomic Integrity and Disease Pathogenesis

**DOI:** 10.3390/ncrna5010011

**Published:** 2019-01-21

**Authors:** Priyanka Barman, Divya Reddy, Sukesh R. Bhaumik

**Affiliations:** Department of Biochemistry and Molecular Biology, Southern Illinois University School of Medicine, Carbondale, IL 62901, USA; pbarman@siu.edu (P.B.); 3024reddy@gmail.com (D.R.)

**Keywords:** antisense transcription, long non-coding RNA, chromatin modification, *GAL10*, NuA4, TFIID, SAGA, RNA polymerase II

## Abstract

Non-coding antisense transcripts arise from the strand opposite the sense strand. Over 70% of the human genome generates non-coding antisense transcripts while less than 2% of the genome codes for proteins. Antisense transcripts and/or the act of antisense transcription regulate gene expression and genome integrity by interfering with sense transcription and modulating histone modifications or DNA methylation. Hence, they have significant pathological and physiological relevance. Indeed, antisense transcripts were found to be associated with various diseases including cancer, diabetes, cardiac and neurodegenerative disorders, and, thus, have promising potentials for prognostic and diagnostic markers and therapeutic development. However, it is not clearly understood how antisense transcription is initiated and epigenetically regulated. Such knowledge would provide new insights into the regulation of antisense transcription, and hence disease pathogenesis with therapeutic development. The recent studies on antisense transcription initiation and its epigenetic regulation, which are limited, are discussed here. Furthermore, we concisely describe how antisense transcription/transcripts regulate gene expression and genome integrity with implications in disease pathogenesis and therapeutic development.

## 1. Introduction

Eukaryotic transcription of the protein-coding genes is a highly coordinated and complex process initiated by an assembly of general transcription factors and RNA Polymerase II at the promoter by an activator protein (activator), followed by elongation, and, finally, termination [1,2,3,4,5,6,7]. This process is tightly regulated by epigenetic factors and processes such as DNA methylation, histone modifications, and/or ATP-dependent chromatin remodeling [8,9,10,11,12,13,14,15,16,17,18,19,20,21,22,23,24,25,26]. These epigenetic events and transcriptions are further controlled by non-coding RNAs that include siRNAs (small interfering RNA), miRNAs (microRNA), piRNAs (Piwi-interacting RNA), lncRNAs (long non-coding RNA) or antisense non-coding transcripts [27,28,29,30,31,32,33,34,35,36,37,38,39,40,41]. Non-coding antisense transcripts are generated from the strand opposite the sense strand and control sense transcription (and, therefore, gene expression). About 72% of the human genome generates antisense transcripts [42,43]. Antisense transcripts have important physiological and pathological significance [27,28,29,30,31,32,33,34,35,36,37,38,39,40,44,45,46,47,48,49,50,51,52,53,54,55,56]. Therefore, there are a number of studies on antisense oligonucleotide-based therapy for the regulation of gene expression, with clinical trials for treatment of various diseases including cancer, hypertension, respiratory illness, neurological and muscular disorders, and HIV infection [57,58,59,60,61,62]. Although antisense transcripts/transcription have great potential in disease pathogenesis and treatment, it remains unclear how antisense transcription is initiated and regulated by the chromatin structure. Such knowledge would provide additional avenues of regulation of antisense transcription/transcripts towards therapeutic development and disease pathogenesis. However, only a limited number of studies have focused on understanding the mechanisms of antisense transcription initiation and its regulation by the chromatin structure. These studies are discussed here. Furthermore, the roles of antisense transcription/transcripts in the regulation of gene expression and genomic stability, with implications in disease pathogenesis and therapeutic development, are also described below.

## 2. Antisense Transcription Initiation

Antisense transcripts were originally identified in bacteria [63]. Later, it was found that antisense transcripts are wide-spread throughout eukaryotic genomes [38,64]. More than 70% of the transcripts in humans and mice have antisense transcripts [42,43]. Antisense transcripts generally have a low abundance [65], and prefer to accumulate in the nucleus [66]. However, some antisense transcripts are found in the cytoplasm and the mitochondria [67]. Antisense transcripts are generated from independent promoters, bidirectional promoters of divergent transcription units or cryptic promoters [68,69,70,71,72,73,74,75,76]. Aside from their antisense orientation, antisense transcripts do not possess specific biochemical characteristics. Generally, antisense transcripts do not code for proteins, since the antisense transcript sequence is constrained by overlapping sense transcripts. However, there are examples of pairs of sense and antisense transcripts overlapping partially and both having protein-coding activity [77,78,79]. Independent of protein-coding potential/activity, antisense transcripts can contain specific domains that can interact with DNA, RNA or proteins to form specific functional complexes to execute cellular activities [80,81,82].

**Figure 1:**
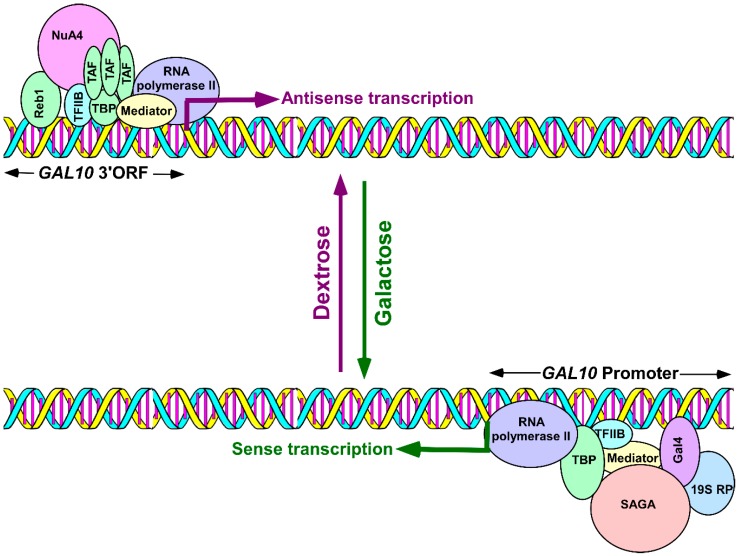
Schematic diagram showing *GAL10* sense and antisense transcriptions in galactose and dextrose-containing growth media, respectively. Bottom panel: The activator, Gal4, targets the co-activator, SAGA, to the *GAL10* upstream activating sequence to enhance the formation of PIC, via the Mediator complex, independent of the TAFs that initiate *GAL10* sense transcription a in galactose-containing growth medium [88,89]. The 19S proteasome subcomplex, or 19S RP, enhances the targeting of SAGA to Gal4 independent of the proteolytic activity of the 26S proteasome [97]. Top panel: Reb1 targets NuA4 KAT to the 3′-end of the *GAL10* coding sequence (*GAL10* 3′ORF) for histone H4 acetylation and targeting of RNA polymerase II, via TFIID, to initiate *GAL10* antisense transcription in dextrose-containing growth medium [83,84]. PIC—pre-initiation complex; SAGA—Spt-Ada-Gcn5-Acetyltransferase; TBP—TATA-box binding proteins; TAFs—TBP-associated factors; NuA4—Nucleosome acetyltransferase of histone H4; TFIID—Transcription factor IID, a complex of TBPs and a set of TAFs; TFIIB—Transcription factor IIB; and 19S RP—19S regulatory particle.

Although antisense transcription is wide-spread throughout eukaryotic genomes, it is not clearly understood how antisense transcription is initiated because it is technically difficult to study the mechanisms of antisense transcription in the background of sense transcription. Recently, we took advantage of the *GAL* gene cluster in yeast to study the mechanisms of antisense transcription initiation in a dextrose-containing growth medium that is permissive to antisense transcription but not to sense transcription [83,84]. *GAL1*, *GAL7*, and *GAL10* constitute the *GAL* gene cluster, a galactose-inducible genetic unit. In this cluster, *GAL1* and *GAL10* are divergent genes with a bidirectional promoter, while *GAL10* and *GAL7* are tandem genes. Such organization has significant implications in gene regulation through transcriptional interference [85]. Previous studies [86,87] reported the existence of 2.6, 4, and 6 kb long non-coding antisense transcripts that initiated from the 3′-end of the *GAL10* coding sequence in a dextrose-containing growth medium that represses *GAL10* sense transcription. Such *GAL10* antisense transcription attenuates *GAL1-GAL10* sense transcription. Using this system, we analyzed the mechanisms of antisense transcription initiation, from the 3′-end of the *GAL10* coding sequence, in a dextrose-containing growth medium [83,84]. *GAL10* antisense transcription was found to be dependent on a Myb-related protein Reb1 that binds to the 3′-end of the *GAL10* coding sequence [83,84,86,87]. The Reb1 binding site is located 158 bp upstream of the TATA-box at the 3′-end of the *GAL10* coding sequence and 380 bp downstream of the translational stop codon [87]. However, there is another TATA-box 221 bp upstream of the Reb1 binding site [87]. Reb1 targets the recruitment of NuA4 (nucleosome acetyltransferase of histone H4) KAT (lysine (K) acetyltransferase) to the 3′-end of the *GAL10* coding sequence for histone H4 acetylation targeting TBPs (TATA-box binding proteins) and TBP-associated factors (TAFs). This forms the pre-initiation complex (PIC) in recruiting RNA polymerase II (Figure 1). Consistently, NuA4 KAT, TBPs, TAFs, TFIIB (Transcription factor IIB) and a Mediator are required for the recruitment of RNA polymerase II to the 3′-end of the *GAL10* coding sequence to initiate antisense transcription [83,84]. Under this growth condition (i.e., a dextrose-containing growth medium), RNA polymerase II, associated with sense transcription, was not found at the 3′-end of the *GAL10* coding sequence [83]. This was because of a zinc finger protein Mig1-mediated repression as well as the masking the Gal4 activation domain by the repressor, Gal80, in a dextrose-containing growth medium [88,89,90,91,92,93,94]. Thus, our results [83,84] demonstrated, for the first time, the roles of various transcription factors, TBPs, TAFs, TFIIB, NuA4 and the Mediator, as well as the activator-binding site or Reb1 in facilitating the recruitment of RNA polymerase II to the antisense transcription initiation site at the 3′-end of the *GAL10* coding sequence for antisense transcription initiation (Figure 1).

Intriguingly, we found that *GAL10* antisense transcription is dependent on TFIID (transcription factor IID; an assembly of TBPs and TAFs), while its sense transcription does not require TFIID (Figure 1) [83,88,89,95,96]. Furthermore, *GAL10* antisense transcription does not depend on the 19S proteasome complex or 19S regulatory particle (19S RP) that is required for sense transcription (Figure 1) [83,97]. Moreover, the Gal4 activator, which is essential for the sense transcription of *GAL10*, is dispensable for *GAL10* antisense transcription (Figure 1) [83,88,89,96]. Furthermore, SAGA (Spt-Ada-Gcn5-acetyl-transferase) is required as a co-activator for *GAL10* sense transcription but is dispensable for *GAL10* antisense transcription (Figure 1) [83,88,89,96]. These results [83,84] supported the idea that *GAL10* sense and antisense transcriptions are independent of each other and are regulated differently (Figure 1). Overall, our results [83,84] provided significant insights into the mechanisms of antisense transcription initiation from the 3′-end of the *GAL10* coding sequence.

The initiation of antisense transcription from other loci might be regulated similarly to that of *GAL10*. However, it would be difficult to study the genome-wide mechanisms of antisense transcription initiation technically as antisense transcription is generally less frequent and occurs in the background of sense transcription. Furthermore, antisense promoters are less characterized and relatively weak. Nonetheless, our studies at the *GAL* locus, in the absence of sense transcription, clearly identified a distinct mechanism of antisense transcription initiation [83,84]. Additionally, it is technically challenging to track RNA polymerase II associated with antisense transcription in the background of sense transcription. Using the *GAL* system, we tracked RNA polymerase II, which is associated with *GAL10* antisense transcription in the absence of sense transcription in a dextrose-containing growth medium [83]. Although our work at the *GAL* locus deciphered the mechanisms of antisense transcription initiation, it is poorly understood how antisense transcription is initiated from cryptic or bidirectional promoters.

## 3. Chromatin Regulation of Antisense Transcription

As DNA is packaged into chromatin within nucleus, chromatin structure is likely to play an important role in regulating antisense transcription. Indeed, our recent studies demonstrated that NuA4 KAT was targeted to the antisense transcription initiation site at the 3′-end of the *GAL10* coding sequence for histone H4 acetylation [84]. Such targeting of NuA4 KAT or histone H4 acetylation promotes antisense transcription initiation by facilitating the recruitment of RNA polymerase II [84], similar to our results for sense transcription of the ribosomal protein genes and *PHO84* [98,99,100,101,102,103]. Like NuA4 KAT, histone H3 K4 methyltransferase (Set1) and histone H3 K36 methyltransferase (Set2), which are required for histone H3 K4 and K36 methylation, respectively, also facilitate antisense transcription from the 3′-end of the *GAL10* coding sequence [84]. Similarly, histone H2B ubiquitylation (which regulates histone H3 K4 methylation [104]) promotes *GAL10* antisense transcription [84]. However, *GAL* sense transcription is facilitated by the histone H2B ubiquitylation independent of histone H3 K4 methylation [105,106]. Thus, the SAGA that possesses histone H2B deubiquitylation activity via its Ubp8 subunit [107] is likely to control antisense transcription. Intriguingly, SAGA was found to be dispensable for antisense transcription from the 3′-end of the *GAL10* coding sequence but is required for the sense transcription of *GAL* genes [84,88,89,96,108,109,110,111]. In agreement with this, SAGA is not recruited to the 3′-end of a *GAL10* coding sequence in a dextrose-containing growth medium [88,96] that is permissive for *GAL10* antisense transcription but not sense transcription. However, in a galactose-containing growth medium that is permissive to *GAL10* sense transcription, SAGA is targeted to the upstream activating sequence of *GAL10* by the activator Gal4 [88,89,96]. Thus, the chromatin modification factor, SAGA, is differentially required for *GAL10* sense and antisense transcriptions. Like SAGA, an ATP-dependent chromatin remodeling factor, SWI/SNF (switching–defective/sucrose non-fermenting) complex, is dispensable for *GAL10* antisense transcription [84], but rather is required for sense transcription [112,113]. However, other ATP-dependent chromatin remodeling factor(s) may be involved in regulation of antisense transcription, which needs to be investigated further. Nonetheless, these recent studies demonstrate the roles of chromatin modification factors on the regulation of *GAL10* antisense transcription. Antisense transcription from other loci is likely to be similarly epigenetically regulated, something which needs to be elucidated further.

## 4. Antisense Transcription in Regulation of Sense Transcription and Chromatin Structure

An antisense transcript can function by itself and/or by the act of its transcription in cis (which controls genes locally on the DNA strand involved in its origination) and/or in trans (which regulates genes on other DNA strands). The trans effect is usually mediated by the antisense transcript, while the cis effect is generally due to the act of antisense transcription [114]. Three-dimensional organization of chromatin can also allow the regions/sites of antisense transcription to interact with other loci for trans effects. Furthermore, an antisense transcript can be present at the place of its synthesis via stalled RNA polymerase, R-loops or triple helices, to exert its function in cis. It is suggested that antisense transcription/transcripts function more frequently in cis than in trans [80].

Antisense transcription regulates sense transcription by affecting DNA methylation at the CpG islands at the promoter [30,115]. For example, the hemoglobin α1 gene (*HBA1*) in α-thalassemia patients is repressed by antisense transcription, where an aberrant LUC7L (putative RNA-binding protein Luc7-like) RNA runs antisense to the *HBA1* locus and methylates the CpG island at the promoter to repress the *HBA1* gene expression [30]. Antisense transcription was also found to be involved in gene imprinting [116,117]. For example, antisense transcription of AIRN (antisense to insulin-like growth factor 2 receptor (IGF2R) non-coding RNA), but not AIRN transcript, represses *IGF2R* through transcriptional interference and DNA methylation in mice [117]. However, antisense transcription can also stimulate sense transcription by inhibiting de novo methylation at the promoter via R-loop formation [118,119]. Sense transcription is also regulated by antisense transcription via histone modifications. For example, X chromosome inactivation occurs through the regulation of histone modification by antisense expression. Antisense transcription also regulates histone modification in trans via antisense transcript. One classic example is mammalian HOTAIR (HOX transcript antisense intergenic RNA) that regulates histone modification, via PRC2 (polycomb repressive complex 2, required for histone H3 K27 methylation and a repressive mark), to control sense transcription [119,120]. In plants, a set of antisense transcripts to *FLC* (flowering locus C), namely COOLAIR (cold-assisted intronic non-coding RNA), increases histone H3 K27 methylation levels through recruitment of polycomb proteins to repress *FLC* expression in response to cold [121]. In addition, another antisense transcript, COLDAIR (cold-induced long antisense intragenic RNA; antisense to COOLAIR), is also responsible for the recruitment of polycomb proteins at the *FLC* locus [122]. In budding yeast, the antisense transcript to inorganic phosphate transporter gene *PHO84* is upregulated upon chronological ageing and represses *PHO84* sense transcription via histone deacetylation [123]. Furthermore, the act of antisense transcription itself regulates chromatin modifications. For example, antisense transcription from the internal cryptic promoters modifies the chromatin of the associated sense genes and, therefore, sense transcription [73,86,87]. In addition to the regulatory mechanisms of sense transcription and chromatin structure by antisense transcription discussed above, the formation of a triple RNA-DNA helix at the promoter in cis and in trans has been implicated in sense transcription repression [124,125,126]. Overall, antisense transcription/transcripts regulate gene expression by promoter methylation [30,117,127], histone modifications [81,128,129,130,131,132,133], or interfering/blocking sense transcriptional machinery [44,124,134,135].

Besides the functions discussed above, antisense transcription also controls mRNA splicing [136,137,138], mRNA stability [46] or translational efficiency through the recruitment of additional factors [139]. Thus, through these activities, antisense transcription/transcripts regulate gene expression. In addition to these gene regulatory functions, antisense transcription/transcripts are also involved in controlling the expression/generation of non-coding RNAs. For example, antisense transcription generates siRNAs from double-stranded sense-antisense hybrids [140,141]. Furthermore, an antisense transcript, namely lncTAM34a, was recently found to modulate the expression of miR34a that is associated with tumor suppression [142]. Thus, antisense transcription/transcripts play important roles in the expression/generation of coding as well as non-coding RNAs.

## 5. Antisense Transcription in Regulation of DNA Repair

In addition to controlling sense transcription, chromatin structure, mRNA splicing and stability, translation, and generation/expression of non-coding RNAs, antisense transcription/transcripts are involved in the regulation of DNA damage response and repair. Cells are continuously attacked by genotoxic factors, and DNA lesions and damage are repaired by various cellular mechanisms, including transcription-coupled DNA repair [143,144,145,146,147,148,149,150,151,152,153,154,155,156]. DNA damage response plays an important role in DNA repair. DNA damage activates checkpoints for cell cycle arrest and DNA repair. If DNA is not repaired or DNA repair fails, apoptosis will be triggered to remove cells with accumulated mutations [157]. Therefore, cell cycle arrest and apoptosis play crucial roles in handling detrimental genotoxic stress. These important processes of DNA repair are regulated by transcription factors and antisense transcripts/transcription, as described below.

A number of studies have indicated that the expression of antisense transcripts is altered in response to DNA damage in order to control downstream gene expression for DNA repair. For example, transcription of an antisense non-coding RNA is induced from the upstream region of the *CCND1* (cyclin D1, a cell cycle regulator) gene in response to genotoxic stress [158]. Such damage-induced antisense transcripts establish a hypo-acetylated chromatin state upon binding to the RNA binding protein TLS (translocated in liposarcoma that inhibits CBP/p300 histone acetyltransferase activity), thus repressing *CCND1* sense expression [159]. The reduced expression of *CCND1* is associated with cell cycle arrest and check point activation for DNA repair. Another example is the long intergenic non-coding RNA-p21 (or lincRNA-p21) that is transcribed from the opposite strand of p21 (*CDKN1A*), a cell cycle regulator, in response to DNA damage [160]. Unlike the antisense RNA at *CCND1* that acts locally, lincRNA-p21 functions globally to repress transcription of the genes that are associated with apoptosis and DNA repair [160]. Another antisense non-coding RNA at the *INK* locus is ANRIL (a 3.8 kb transcript in the opposite orientation of *INK4B*-*ARF*-*INK4A* gene cluster; also known as CDKN2B-AS1). This is also induced by the transcription factor E2F1 (E2F transcription factor 1) in an ATM (ataxia-telangiectasia mutated)-dependent fashion in response to DNA damage [161]. The transcriptional induction of ANRIL reduces the expression of *INK4A* (also known as *CDKN2A* and p16), *INK4B* (also known as *CDKN2B* and p15) and *ARF* (alternate reading frame; also known as p14) [162]. Such altered transcription of *INK4A*, *INK4B* and *ARF* permits cells to go back to their normal state following DNA repair, via the impediment of cell cycle checkpoints and stimulation of cell cycle progression [162]. In addition, ANRIL is also associated with homologous recombination-mediated DNA repair pathways [162]. Another antisense RNA, known as DLX6-AS1 (DLX6 antisense RNA 1 or Evf2 lncRNA), is involved in the regulation of DNA repair via interaction with the catalytic BRG1 (Brahma-related gene 1) subunit of the SWI/SNF chromatin remodeling complex through its ATM-dependent phosphorylation. Loss of BRG1 is associated with impaired homologous recombination [163,164,165]. Another antisense RNA, PANDA (p21-associated non-coding RNA, DNA damage activated) is located 4.5 kb upstream of the cell cycle regulator *CDKN1A* (p21) transcriptional start site. It is also induced in response to p53-dependent DNA damage [166]. PANDA interacts with the transcription factor NF-YA and suppresses the transcription of pro-apoptotic genes [166]. Thus, DNA damage induced PANDA prevents apoptosis via the recruitment of NF-YA. Furthermore, PANDA stabilizes p53 in response to DNA damage. Likewise, there are many examples of antisense transcripts associated with DNA repair [167,168,169]. Thus, antisense transcription/transcripts play important roles in the regulation of genomic integrity. Misregulation of antisense transcription/transcripts would alter genomic integrity, leading to cellular pathologies.

## 6. Antisense Transcription in Cancer

Antisense transcription/transcripts represent potential prognostic and diagnostic markers for therapeutic development for cancer. A promising candidate, HOTAIR, is significantly overexpressed in multiple tumors, including breast, colorectal, hepatocellular and pancreatic cancers [119,170,171,172,173,174,175,176]. HOTAIR is a 2.2 kb lncRNA that originates from the *HOXC* locus, antisense to the *HOXC11* and *HOXC12* genes [170]. This antisense RNA was found to interact with PRC2 for histone H3 K27 methylation and to silence chromatin [119]. HOTAIR enhances the occupancy of PRC2 at the *HOXD* locus, and silences transcription of the *HOXD* genes by altering the chromatin structure. Overexpression of HOTAIR induces genome-wide re-targeting of PRC2 to several hundred genes, leading to altered histone H3 K27 methylation, cancer progression and malignancy [119,170,171,172,173,174,175,176]. Importantly, the knockout of HOTAIR inhibits cell proliferation and migration, and induces apoptosis and cell cycle arrest in various cancer types [119,170,171,172,173,174,175,176]. Another widely studied antisense RNA is H19, one that is transcribed from *H19/IGF2* on chromosome 11. Its overexpression is linked to cellular migration and invasion in various cancers including stomach, breast, liver, lung, and pancreas cancers [177]. Yoshimura et al. [178] reported that the inhibition of H19 antisense RNA could be an effective therapeutic strategy for the treatment of pancreatic cancer. Recently, another antisense RNA, MAPT-AS1 (MAPT antisense RNA 1), has been reported as a potential therapeutic target in ER (estrogen receptor)-negative breast cancers [179]. MAPT-AS1 was found to be highly expressed in breast cancer cells. Upregulation of this antisense RNA is associated with metastasis in breast cancer and other cancers, while its depletion reduces the proliferation and migration of cancer cells, thus implicating MAPT-AS1 as a therapeutic target for the treatment of ER-negative breast cancers [179].

There are other antisense RNAs that could be potential targets for cancer therapy. These include WRAP53 (WD repeat containing antisense to TP53), HOXA-AS2 (*HOXA* cluster antisense RNA 2), HOXA11-AS (*HOXA11* Antisense RNA), PANDA, and ANRIL. WRAP53 regulates the tumor suppressor p53 and is overexpressed in a variety of tumor cell lines [180]. HOXA-AS2 is upregulated in breast cancer and its silencing inhibits the progression of breast cancer [181]. Thus, HOXA-AS2 can be a potential prognostic marker and therapeutic target for breast cancer. HOXA11-AS is upregulated in human gastric cancer cells [182]. PANDA is upregulated in gastric and breast cancers, and downregulated in non-small cell lung cancers [183,184,185]. Likewise, ANRIL is highly expressed in cancers including non-small cell lung cancer and cervical cancer, its depletion inhibits cell proliferation [186,187]. In addition to these antisense transcripts, there are other antisense transcripts involved in various cancers [188,189,190,191,192,193,194,195,196]; thus, it could serve as potential biomarkers and/or therapeutic targets for cancer therapy. Furthermore, a comprehensive dataset has been generated for a positive correlation of the differential expressions of sense–antisense transcripts with cancer [197,198].

## 7. Antisense Transcription in Neurological Disorders

In addition to their association with cancer, antisense transcripts are also involved in neurological disorders. The characterization of these transcripts and their modes of action may allow them to be used for diagnosis, monitoring disease progression and targeted therapies in neurological disorders. One important antisense transcript, BACE1-AS (*β*-site amyloid precursor protein-cleaving enzyme-antisense), is associated with Alzheimer’s disease. BACE1-AS is a 2 kb long transcript originating from *BACE1* (*β* secretase 1) in the antisense orientation [46]. This antisense transcript plays an important role in enhancing the stability of BACE1 mRNA via the formation of the RNA duplex and, thus, leading to the elevated levels of BACE1 protein that are essential for the generation of β-amyloid [199]. The knockdown of this antisense transcript decreases the level of BACE1, thus reducing amyloid formation and aggregation in the brain. In Alzheimer’s disease, BACE1-AS is highly expressed and promotes amyloid formation via the enhanced stability of BACE1 [200], implicating BACE1-AS as an important biomarker and potential therapeutic target for the treatment of Alzheimer’s disease. Another antisense RNA, known as UCHL1-AS (ubiquitin carboxy-terminal hydrolase L1-antisense), is a 1.2 kb lncRNA that targets UCHL1 mRNA to heavy polysomes for efficient translation and to enhance UCHL1 protein level [139]. Overexpression of this UCHL1 was found to be associated with reduced amyloid β production and the delayed progression of Alzheimer’s disease [201]. Both UCHL1 and UCHL1-AS are downregulated in Parkinson’s disease [202]. Another antisense transcript, PINK1-AS (*PINK1* antisense RNA), is transcribed from the antisense direction of the *PINK1* gene that encodes PTEN (phosphatase and tensin homologue deleted on chromosome 10)-induced putative kinase 1. Mutation in the *PINK* locus causes the early onset of Parkinson’s disease. The PINK1-AS stabilizes the expression of a PINK1 splice variant, svPINK1, via a double strand RNA-mediated mechanism [203]. The silencing of PINK1-AS results in the reduced expression of svPINK1 in neuronal cells [203]. Thus, the modulation of the PINK1-AS expression may have a direct impact on Parkinson’s disease. Huntington’s disease (HD) is also associated with an antisense transcript, HTTAS (Huntingtin antisense). HTTAS is a natural antisense transcript at the Huntingtin CAG trinucleotide repeat locus. It is alternatively spliced into HTTAS-v1 (exons 1 and 3) and HTTAS-v2 (exons 2 and 3). Using cell systems, the HTTAS-v1 overexpression was found to correlate with reduced endogenous transcript levels of HTT (Huntingtin), while the knockdown of HTTAS-v1 positively influenced the HTT transcript level. The reduced expression of HTTAS-v1 was observed clinically in human HD frontal cortexes, suggesting the involvement of HTTAS-v1 in the regulation of HTT expression and the progression of HD [204,205]. Another antisense lncRNA, TUG1 (Taurine upregulated gene 1), is upregulated in HD patients [205]. TUG1 interacts with the EZH2 (enhancer of zeste homolog 2) component of PRC2 and, thus, epigenetically represses the expressions of the target genes [206,207]. The depletion of TUG1 induces apoptosis [208]. Thus, the dysregulation of TUG1 is associated with HD and other neurological disorders [205]. Likewise, there are other antisense transcripts associated with neurological disorders [209,210,211,212,213,214].

## 8. Antisense Transcription in Diabetes, Cardiovascular and Other Diseases

In addition to their involvement in cancer and neurological disorders, antisense transcripts are also associated with various other diseases including diabetes and cardiovascular disorders. Transcriptome-wide studies have revealed several antisense transcripts to be involved in diabetes mellitus, a metabolic disorder associated with high blood glucose levels. Misregulation of these antisense transcript expressions is linked to both type 1 and type 2 diabetes [215,216,217,218,219]. The antisense transcript ANRIL is a hot spot region associated with type 2 diabetes, diabetic nephropathy, diabetic retinopathy, diabetic cardiomyopathy and coronary artery disease (CAD). ANRIL expression is high in retina and retinal endothelial cells due to hyperglycemia. The elevated expression of ANRIL has been shown to regulate the transcription and function of VEGF (vascular endothelial growth factor) via interaction with epigenetic factors, namely p300 and PRC2, in diabetic mice [215]. The knockout of ANRIL in mice resulted in a low level of VEGF, as well as extracellular matrix proteins [215]. Thus, ANRIL controls the heart, kidneys and eyes in diabetes by regulating the expression of VEGF and the extracellular matrix proteins [215]. These findings suggest a novel therapeutic strategy to control diabetes and associated complications using an RNA-based approach. Furthermore, Qiu et al. [216] identified another antisense lncRNA, MEG3 (maternally expressed gene 3), in microvascular dysfunction, an important feature in diabetes complications [216]. In this study, the expression level of MEG3 was found to be significantly low in the retinas of streptozotocin-induced diabetic mice and in endothelial cells under high glucose stress. The knockdown of MEG3 significantly exacerbated retinal vascular abnormalities, resulting in endothelial cell proliferation, migration and tube formation [216]. Thus, MEG3 upregulation may serve as a new therapeutic approach in the treatment of diabetes-induced microvascular complications. Likewise, there are many antisense transcripts that are significantly misregulated in diabetes and its associated complications [217,218,219].

In addition to the involvement of antisense RNAs in cardiac diseases via diabetes, antisense transcription/transcripts are also directly associated with cardiovascular diseases. Recent studies have implicated antisense RNAs as new diagnostic markers with therapeutic potentials for cardiovascular diseases [210,211,212,220,221,222,223,224,225,226,227,228,229,230,231,232,233,234,235,236,237,238,239]. For example, an antisense transcript H19, discussed above, is associated with cardiovascular diseases such as CAD. The knockdown of H19 induces cardiomyocyte hypertrophy [220], indicating H19′s role in maintaining cardiac health. Furthermore, H19 functions as a precursor for miR-675, which inhibits cardiac hypertrophy [221]. Importantly, CaMKIId (calcium/calmodulin-dependent protein kinase II delta), a downstream target of H19-miR-675, is a serine/threonine protein kinase that is associated with cardiac electrical conduction. Thus, misregulation of CaMKIId by H19-miR-675 has been linked to cardiac electrical conduction defects. Furthermore, H19 was found to be upregulated in atherosclerosis and to increase the levels of H19 in VSMC (vascular smooth muscle cells) and HUVSC (human umbilical vein endothelial cells), resulting in cellular proliferation and the suppression of apoptosis [222]. Similarly, another antisense RNA, MALAT1 (metastasis-associated lung adenocarcinoma transcript 1), is linked to cardiovascular diseases including hypertension and diabetic cardiomyopathy [223,224]. In addition, MALAT1 has been demonstrated to play a regulatory role in promoting angiogenesis via VEGFR2 (vascular endothelial growth factor receptor 2) [225]. Likewise, ANRIL is also associated with cardiac diseases including myocardial infarction (MI) and CAD. The promoter of ANRIL was found to be methylated in individuals with a high risk of CAD [226,227]. ANRIL variants (e.g., rs10965215 and rs10738605) resulting from single nucleotide polymorphism (SNP) are associated with the risk of MI in the Chinese Han population [228]. The ANRIL variant rs3217992 has been connected to adverse cardiovascular events [229]. Furthermore, another ANRIL SNP variant, rs10757278, was linked to the development of major adverse cardiovascular events in hemodialysis patients [230]. The expression level of the ANRIL transcript was found to be remarkably low in the blood of CAD patients, indicating a relationship between the expression level of ANRIL and the risk of disease. Like ANRIL, HOTAIR is also involved in AMI (acute MI) and its plasma concentration could be used to detect and monitor AMI [231]. HOTAIR is upregulated in the cardiac tissues and plasma of patients with AMI and CAD [231,232]. Together, these studies demonstrate the roles of antisense transcripts in the regulation of cardiac health and functions. These antisense transcripts could be diagnostic markers and therapeutic targets for cardiovascular diseases. In addition, many other antisense transcripts are correlated with cardiovascular diseases [210,211,212,233,234,235,236,237,238,239].

Antisense transcripts are also involved in regulating muscular diseases. For example, MALAT1 is linked to muscular dystrophy [240,241]. In proliferating myoblasts, MALAT1 recruits *SUV39H1* (Su(var)3-9 homolog 1) to the binding site of the muscle differentiation regulator MyoD, and causes trimethylation of the histone H3 K9, thus repressing the expression of the MyoD target genes involved in muscle differentiation [240]. On the other hand, the knockdown of *MALAT1* promotes myogenic differentiation in cultured cells [240]. In agreement with this, increased muscle regeneration was observed in *MALAT1* knockout mice [240]. Recently, MALAT1 was also reported as a novel downstream target of myostatin [241], a negative regulator of muscle growth [242]. Thus, MALAT1 is associated with the regulation of myogenic differentiation and muscle regeneration [240], and the misregulation of MALAT1 is linked to muscular disorders [240,241]. Another antisense lncRNA, SIRT1 AS (sirtuin 1 antisense RNA), has also been shown to regulate myogenesis. The knockdown of SIRT1 leads to the differentiation of myoblasts in C2C12 and human skeletal muscle cells. The overexpression of SIRT1 AS increases the levels of the NAD^+^-dependent histone/protein deacetylase, SIRT1, via the formation of RNA duplexes and the facilitation of SIRT1 translation, by competing with miR34a (that can bind with SIRT1) to inhibit muscle formation [243,244]. Thus, dysfunction or misregulation of SIRT1 AS would alter myogenesis, leading to muscular diseases. Likewise, there are other antisense RNAs involved in muscle diseases [210,212,245,246]. Antisense transcripts were found to be involved in immune diseases [247,248,249]. For example, H19 is upregulated in rheumatoid arthritis patients [247]. HOTAIR was found to be expressed at high levels in rheumatoid arthritis [248]. MALAT1 is also overexpressed in rheumatoid arthritis fibroblast-like synoviocytes [249]. Thus, antisense transcripts have the potential to function as biomarkers in immune diseases.

Since gene expression is central to cellular processes, the regulation of protein-coding gene expression by antisense transcription/transcripts has a significant impact on cellular gene expression and health. The misregulation of antisense transcription or antisense transcripts would be associated with various diseases, some of which are discussed above. However, other diseases/conditions such as aging, metabolic disorders, stress, thalassemia and spinocerebellar ataxia are also associated with antisense transcription/transcripts [30,210,211,212].

## 9. Conclusions

Antisense transcripts are wide-spread throughout eukaryotic genomes and are generated from independent, bidirectional or cryptic promoters. Antisense transcription/transcripts regulate gene expression and genome integrity via transcriptional interference, histone modification, and/or DNA methylation. Antisense transcripts can bring different macromolecules together within the three-dimensional context of the cell to coordinately execute transcriptional, post-transcriptional, and epigenetic processes. Thus, antisense transcription/transcripts are involved in many biological processes and are misregulated in a variety of diseases including cancer, neurological diseases, diabetes and cardiovascular disorders. Therefore, an understanding of the regulatory mechanisms of antisense transcription, characterization of antisense transcripts and their modes of actions would be useful for the diagnosis, monitoring and targeted therapies of various diseases. Indeed, several antisense oligonucleotides are in clinical trials for the treatment of various diseases [62]. Two antisense oligonucleotide-mediated therapies are now available in clinics for the treatment of Duchenne muscular dystrophy and spinal muscular atrophy [62]. Thus, the rapid development of antisense transcription-based therapy holds great promise for the treatment of many diseases in the near future.

Although antisense transcription/transcripts play crucial roles in the regulation of gene expression and genomic integrity due to their involvement in various diseases, it remains unclear how antisense transcription is initiated and epigenetically regulated. Here, we began to develop an understanding of how antisense transcription is initiated and regulated by histone covalent modifications. Further studies are needed for a thorough understanding of antisense transcription initiation and its epigenetic regulation. Such knowledge will provide new insights into the regulation of antisense transcription/transcripts and will aid in understanding the etiologies of various diseases, therefore promoting the discovery of diagnostic markers and therapeutic interventions.
